# Micro-Mechanisms of Shear Deformation Localization of Ti6Al4V Alloy under Shear-Compressive Loading Conditions

**DOI:** 10.3390/ma13245646

**Published:** 2020-12-10

**Authors:** Lintao Li, Tao Jin, Fei Shuang, Zhiqiang Li, Zhihua Wang, Wei Ma

**Affiliations:** 1 Institute of Application Mechanics, Taiyuan University of Technology, Taiyuan 030024, China; lintao_tyut@163.com (L.L.); jintao@tyut.edu.cn (T.J.); lizhiqiang@tyut.edu.cn (Z.L.); watwm@imech.ac.cn (W.M.); 2 Institute of Mechanics, Chinese Academy of Sciences, Beijing 100190, China; shuangfei@ufl.edu; 3 Department of Mechanical and Aerospace Engineering, University of Florida, Gainesville, FL 32611-6250, USA

**Keywords:** titanium alloy, deformation localization, microcosmic mechanism, shear-compression specimen, dynamic combined loading

## Abstract

Titanium Ti6Al4V alloy is a superior material that has extremely high strength, hardness and good anti-corrosion resistance. Dynamic shear-compression experiments were carried out on the alloy to investigate the micro-mechanisms of adiabatic shear banding (ASB) formation. The split Hopkinson pressure bar (SHPB) setup were used for the tests at high strain rates. It was found that the shear deformation localization (SDL) was considerably affected by the complex loading conditions. The micro-mechanisms for the ASB formation relied on different shear compressive proportion of loadings (SCLPs). Scanning electron microscope (SEM) observations showed that the ASB width was related with the SCLP and the fracture failure of alloy was induced by the nucleation and growth of microvoids. In transmission electron microscope (TEM) analysis, the microstructural changes of material within the ASB were characterized by dynamic recrystallization (DRX) and twining grain formation, dislocation migration, and stacking and grain refining processes. The results in this article demonstrates a complex image of microstructural evolution of alloy in the shear localization process.

## 1. Introduction

Shear deformation localization (SDL) of ductile metals indicates that the concentration of deformation is an outcome of material response. As a mechanism of plastic deformation, such localization is of significance as a precursor to final fracture of materials. When the plastic deformation instantly takes place in a narrow band, in which high strain rate and temperature rise develop, such failure mode is usually referred to as adiabatic shear band (ASB) and is considered as a plastic instability process [1,2,3]. Usually, it is difficult to predict the onset of ASB due to the instantaneity of deformation, and for a long time, a commonly accepted consequence is that the ASB formation relies on the competition between the strain hardening and thermal softening of materials during plastic flow [4,5,6,7,8]. In addition, the researches on the microscopic mechanisms demonstrates that the microstructures and crystallographic properties of materials have significant influence on the ASB formation [9,10,11,12].

The titanium alloy (Ti6Al4V) has a prominent inclination to fail through the ASB formation covering other failure modes. Current investigations in terms of thermoplastic instability theory has come up with the accepted viewpoint that thermal perturbation in plastic flow triggers the ASB nucleation and growth [5,6,8]. However, the up-to-date study on the formation mechanism of ASB has broken through the limitation of thermoplastic instability. The dynamic tests with shear-compression specimen (SCS) demonstrated that dynamic recrystallization (DRX) precedes and triggers ASB onset instead of the temperature rise resulting in thermal softening of material [9,10,11,12]. The latest experimental observation also showed that the high temperature is not the cause of ASB formation since the temperature rise occurs quite latter than the ASB initiation [13]. Additionally, microscopic experiments revealed that the metallurgical characteristics and microstructure of the alloy have obviously changed in the SDL process. For example, the high strength shear stress induces the phase transformation of α-phase and β-phase of alloy [11,14], high strain rate deformation leads to the formation of high density dislocation structure [15,16,17], severe shear strain results in the generations of deformation twinning [18,19,20,21] and the formation of DXR grains [11,12,22]. In the SDL, heavily deformed coarse grains with large size changed into the nanoscale grains or ultrafine grains which provide more slip systems in co-operation for the ASB formation [23,24,25].

In the studies of the failure mechanisms of Ti6Al4V alloy, two kinds of tests were used in complex stress loading environments. One is the dynamic experiment under the shear-compression loading (SCL) conditions completed by Rittel et al. [10,11]. By the transmission electron microscope (TEM) analysis, a large number of DRX nanograins were observed within the ASB of the unbroken specimens. This means that, during the ASB formation, the DRX process essentially preceded the thermal softening effect of material induced by high local temperature rise as generally acknowledged. In order to clarify whether the ASB formation is a dynamic instability process governed by microstructural evolutions, Landau et al. [12] completed large shear deformed tests on the alloy SCSs in which the peak shear strain is near 90% failure strain. When the ASB development induces a large number of microcracks in the gauge fillet of unbroken specimens, the massive DRX nanograins were observed in the interconnecting regions between the microcracks. Outside the severe shear deformation region, heavily deformed subgrains and twining grains, but non DRX grains, were observed. This microstructural characteristic clearly demonstrates that the ASB formation is a plastic instability process caused by the microstructural evolution through the nucleation and growth of DRX nanograins.

Another one is the equal channel angular pressing (ECAP) test as a processing mean for grain refinement under quasi-static loading condicions [15,16,17,18,19,20,21,22,23]. By TEM analysis, Chen et al. [15] showed that, after the ECAE processing, a large number of twining grains formed in the SDL region and the twining deformation plane is the crystal plane {10¯11}. The hcp structure of alloy has fewer slip systems, and thus the twinning deformation controls the plastic flow process. The subgrain formation confirms the occurrence of the continuous DRX process. The TEM analysis of Kim et al. [16] revealed that the formation of the ultrafine grains in the band is a dynamic recovery process, by which the high density of dislocations resutls in the formation of equiaxed subgrains. Then, the continuous DRX process makes the elongated grains convert into the equiaxed crystallites. Chen et al. [21] also observed that the DRX process is a recovery dominated process and proceeds by continuous absorption of dislocations in the low angle grain boundaries of subgrains. This eventually results in the formation of new grains with high angle grain boundaries. Gunderov et al. [22] showed that the microstructure of the alloy is characterized by a high fraction of low angle boundaries and high dislocation density. The deformation twinning results in the formation of elongated grains and the SCP in the ECAP process induces the severe accommodation of shear strain in the alloy.

Above reviews show that, in the quasi-static ECAP case, the ASB formation is related to many microscopic mechanisms as the DRX, twining, dislocation, phase transformation, grain refining and nanograins. Under the dynamic SCL conditions, sufficient evidences showed that the DRX is the dominant mechanism resulting in the ASB formation, but not the traditional thermoplastic instability induced by the perturbation of homogeneous temperature fields. In this case, on more attention is paid to the influences of strain rate sensitivity and proportional loading conditions. These issues are still unclear presently and will be addressed in this work. Dynamic shear-compression tests on the Ti6Al4V alloy was first conducted on the split Hopkinson pressure bar (SHPB) setup. The application of the SCS contrasts a shear deformation of specimen material in a pre-designed gauge region. Then, post-mortem microscopic analysis was performed by using scanning electron microscope (SEM, JSM-7800F, JEOL, Tokyo, Japan) and TEM (TEM, JEM-2100, JEOL, Tokyo, Japan). The aim is not only to clarify the micro-mechanisms of the ASB formation, but the influence of loading conditions on the ASB evolution.

## 2. Experimental Procedure

**Figure 1:**
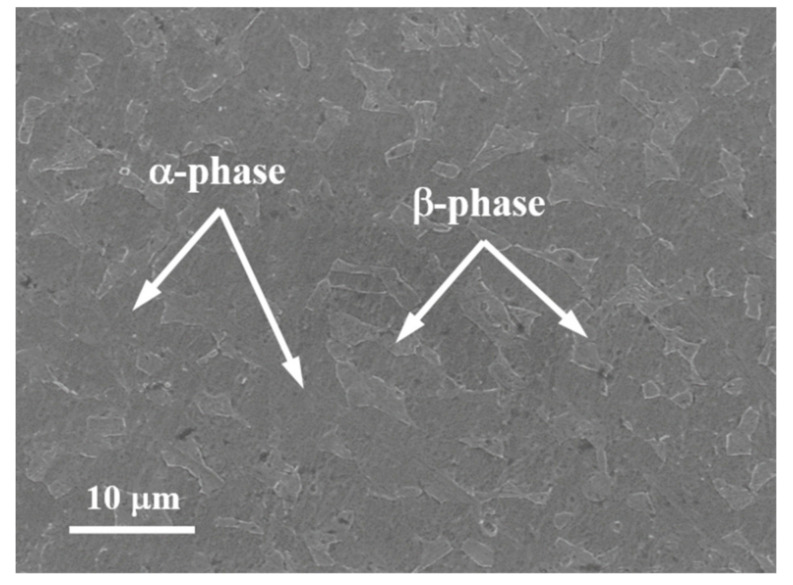
The microstructure of as-received hot rolled cylindrical rods of Ti6Al4V alloy.

The Ti6Al4V alloy used in this test was a hot rolled cylindrical rods of 10 mm in diameter and its chemical composition was Al-6.2, V-4.3, Zr-0.02, Si-0.039, Fe-0.016, O-0.168, C-0.06, N-0.0115, H-0.003, and Ti-balance (wt.%). The presence of bit amounts of ferrum, oxygen, and carbon in the alloy contributes to stabilizing and strengthening the α-phase which is beneficial for improving hardenability, increasing strength and enhancing overall response of the alloy to external applied loads. The homogenerous microstructures of as-received alloy are obtained by heat treatments. The preprocessing conditions are solution treated at 790 °C for 70 min in a protective Argon atmosphere and then naturally cooling in air. Aging treatments are conducted on the solution treated rods at 560 °C for 500 min under ambient air cooled. The as-received material rods has the equiaxed grains both in the center and at the periphery of the bar (Figure 1). The duplex microstructure of alloy consists of the near equiaxed α-phase and transformed β-phase and the abundant β-phase evenly distributes in the primary α-phase matrix. The dimensions of the α- and β-phases were 10–15 µm and the 5–8 µm, respectively. The size of contiguous primary α domains is similar to that of the secondary α + β domains.

**Figure 2:**
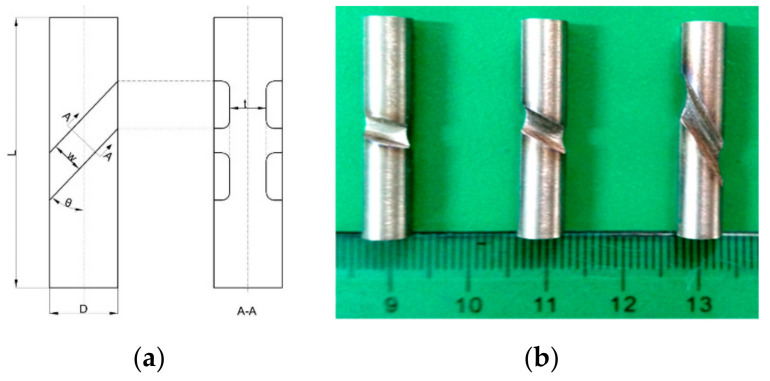
(**a**) The shear-compression specimen (SCS) schematic representation and its dimensions *D* = 6 mm, *L* = 20 mm, *t* = 2 mm *w* = 3 mm, and *θ* = 15°, 30°, and 60° respectively, and (**b**) the photographs of practical specimens.

The SCS of Ti6Al4V alloy used in this test was designed as a cylindrical bar of 6 mm in diameter which was first cut from raw material rods by turning and then a pair of diametrically opposed grooves were machined on the cylinders by using electrical discharge machining. The real specimens and their sizes are shown in Figure 2. The dimensions of SCS were 6 mm in diameter and 20 mm in height. The dimensions of the gooves are 3 mm in width and 2 mm in height. To investigate the effects of different proportional loadings on the deformation localization mechanisms, the inclined angles of the grooves with respect to the longitudinal axis of specimens are selected as *θ* = 15°, 30°, and 60°, respectively. Thus, three loading conditions with different proportions of shear stress to compressive stress, i.e., the SCLPs were obtained for testing. The values of the SCLP are respectively 1.73, 0.58, and 0.27 corresponding to these angles of 15°, 30°, and 60°. The various angles make the specimen geometry and loading conditions determine the deformation localization behavior of material in the grooves since a concentration of stress gives priority to development of strain inside the grooves as the gauge section. Therefore, such a designed SCS has a noticeable inclination of shear failure by ASB formation in the gooves [10,11].

**Figure 3:**
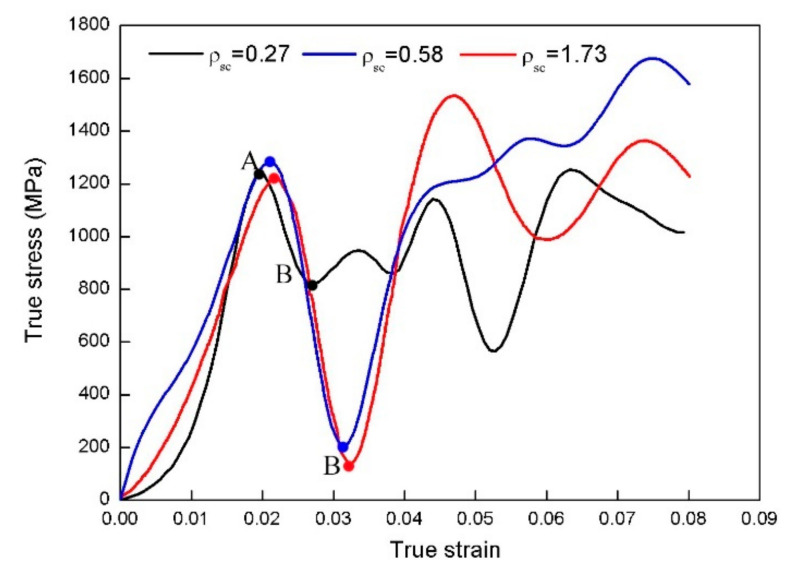
Typical true stress-strain curves obtained in the shear-compressive impacting tests.

The dynamic test is carried out on a SHPB arrangement at a typical strain rate of 3 × 10^3^ s^−1^. Two kinds of tests were conducted: (i) The specimen deformation is free. The impact loading allows the specimen to continuously deform until it is broken by the ASB evolution and the subsequent fracture by mode II crack propagation (ii) The deformation is constrained with a hardened steel stoprings. This constraint allows the specimen to undergo sufficient large shear deformation in the direction of ASB formation, but limited the peak strain of specimen to be less than the fracture strain. The impacting tests designed in this way make different proportional loadings bring the onset and growth of shear bands and will not result in the specimen to break. In the impacting tests with the SHPB setup, elastic stress waves spread in the incident and transmitter bars. From the pulse signals recorded, the typical true stress–strain curves are obtained (Figure 3). The SCLP values produced by the stress waves will rely on the inclined angles between the grooves and the longitudinal axis of specimens.

After the impact tests, the metallographic samples were made for the microstructural analysis. The broken SCSs were polished to a mirror finish and etched with Kroll’s reagent 2% (volume) HF, 8% HNO_3_ and 90% H_2_O for the SEM analysis of fracture mechanism. For the TEM analysis of the micro-mechanisms of ASB formation, the thin foils were prepared by punching out 100 µm thick wafers of 3 mm diameter from the recovered sample and then electrical polishing to perforation in a 7% perchloric acid, 33% butoxy-ethanol and 60% methanal solution. The selection of these foils should include the regions inside and outside the ASB.

## 3. Results and Discussions

Figure 3 exhibits the true stress–strain curves obtained from unbroken SCSs; here, the first peak stress (Point A) corresponds to the onset of shear localization and its initial strain is in the range of 0.02–0.025. The first valley stress (Point B) obtained upon the initial contact of the SCSs with the steel stoprings and the discrete data at point B indicate that the contact between these components occurs at different times. The pre-set gaps between the components ensure shear localization during plastic flow. The irregular fluctuations of these curves after point B demonstrate multiple-contact processes between the SCSs and stoprings and the complex propagation of stress waves in the specimens. The continuous increase in flow stress indicates that the SCSs and stoprings simultaneously get into the subsequent bearing process.

**Figure 4:**
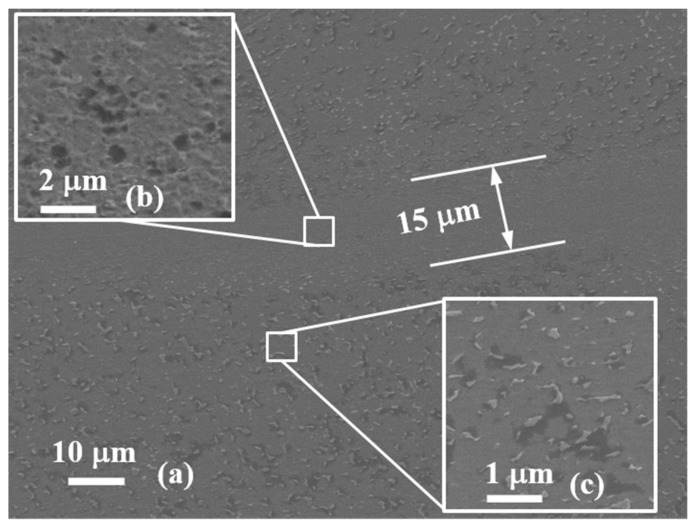
SEM microscopic observation of adiabatic shear banding (ASB) of the specimen with the shear compressive proportion of loadins (SCLP) = 0.58. (**a**) The full morphology of ASB microstructure, (**b**,**c**) are the magnification observations inside and outside ASB.

Figure 4a shows the typical SEM observation on the centrosymmetric surface of the non-fractured specimen and the SCLP is of 0.58. It can be seen that the ASB width is about 15 µm and an evident transition zone of 5 µm in width presents between the SDL band and the uniform deformation region. Inside the ASB, the β-phase almost completely disappeared due to the high temperature rise generated by the severe SDL (Figure 4b). The evolution of the microstructure just gets into the initial nucleation stage of lots of microvoids, but has not arrived in full growth stage. Outside the band, the density of β-phase is evidently decreased comparing with the as-received microstructure of alloy. The β-phase average size has reduced from the order of 10 µm to that of 100 nm (Figure 4c). This implies a loss of material in strength and hardenability, and a degradation of bearing capacity to external loadings.

**Figure 5:**
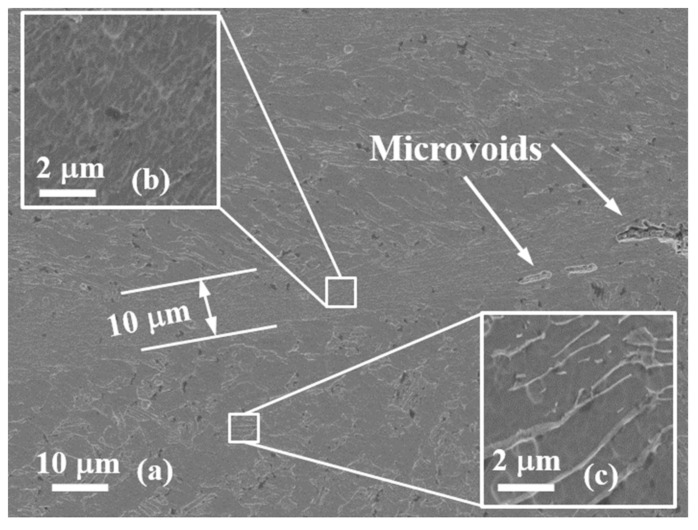
SEM microscopic observation of ASB of the specimen with the SCLP = 1.73. (**a**) The full morphology of ASB microstructure, (**b**,**c**) are the magnification observations inside and outside ASB.

Figure 5 shows the SEM observations of the specimen with the SCLP 1.73. The loading condition with large SCLP value leads to more severe SDL than that with small SCLP (Figure 4). This can be characterized by the decrease of ASB width from 15 µm to 10 µm and the disappearance of the transition zone. The rich filamentous structures in the band demonstrates that the materials subjected to the severe shear deformation. A few of microvoids are still in the initial nucleation stage (Figure 5b), while some microvoids have been fully evolved and formed microcracks (as indicated by the white arrow in Figure 5a). Outside the band, the loading with larger SCLP makes a great deal of large equiaxed grains of β-phase become elongated filamentous grains. Comparing with the results shown in Figure 4 indicates that the small SCLP induces the decrease of material strength by breaking the β-phase grains, while the large SCPL is by elongating the β-phase grains. In both cases, the density of the β-phase outside the band sharply decreases and inside the band the β-phase grains completely disappear. This means that the β-phase of Ti6Al4V alloy is the primary resistance factor in the process of restraining the SDL. Once the β-phase grains are completely consumed during the shear banding formation, the nucleation, growth and cohesion of microvoids begin to proceed one after another, and eventually macro-crack formation results in the failure of material.

**Figure 6:**
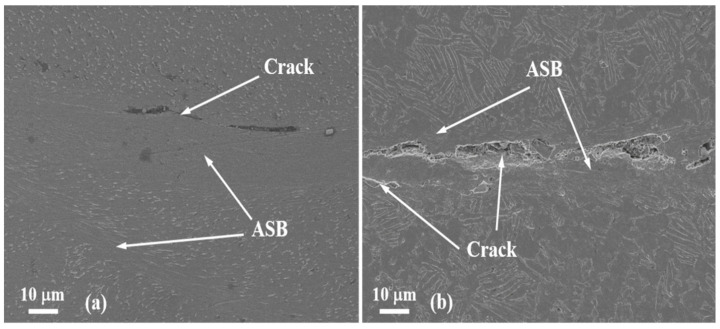
The SEM observations of the crack morphologies in the bands for two specimens with the SCLP are 0.58 (**a**) and 1.73 (**b**) respectively.

As a kind of failure mechanism, the ASB should be developed first in the Ti6Al4V alloy subjected to strong impact loading. After the shear band is fully evolved, the initiation and growth of cracks will result in uncontrolled fracture of material if the external loadings provide enough driving force. Figure 6 shows the SEM observations of the crack growth in the bands for two kinds of specimens of which the SCLP are 0.58 and 1.73 respectively. The cracks developing in the vicinity of ASB are related to the inclining angles. As the SCLP equals 0.58, only one main crack develops in the band and no obvious material damage takes place. However, the formation of a secondary shear band causes the bifurcation phenomenon of ASBs (Figure 6a). As the SCLP equals 1.73, the fracture mechanisms is caused by lots of microscopic cracks and the damage of material in the ASB is evident (Figure 6b). The evolution of many microcracks induces the bifurcation phenomenon of crack propagation in the band. The development of massive microvoids indicates that the crack formation attributes to the severe damage mechanism of material, that is, it has undergone the processes of the nucleation and growth of microvoids as well as the extension and cohesion of microscopic cracks. The density of microvoids is evidently larger in the specimen with the large SCLP than that with the small one, implying that the failure behavior of the alloy obviously tends to the fracture of mode II crack.

**Figure 7:**
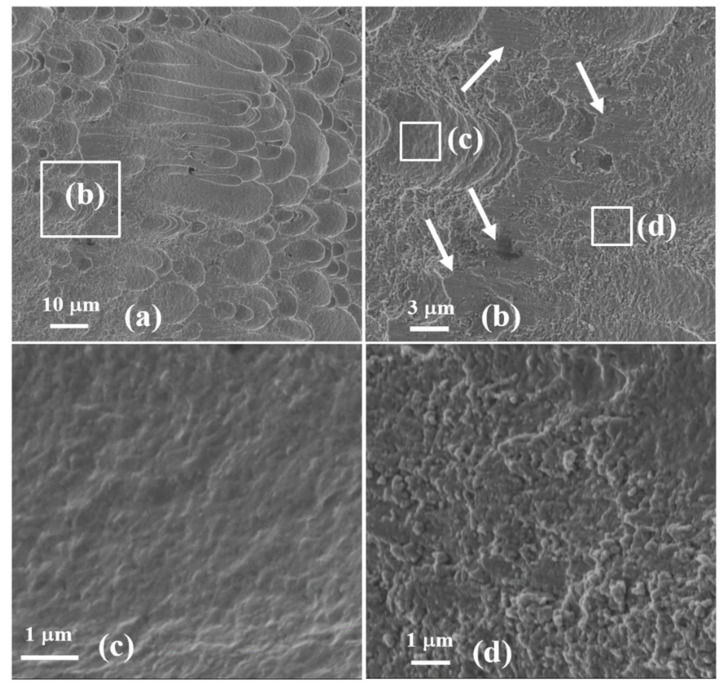
The morphology of the fracture surface in the specimen with the SCLP of 0.58. (**a**) The overall microstructural observation of the crack surface; (**b**) a high magnification of the crack surface, and the further observations with higher magnification inside (**c**) and outside (**d**) the dimple.

**Figure 8:**
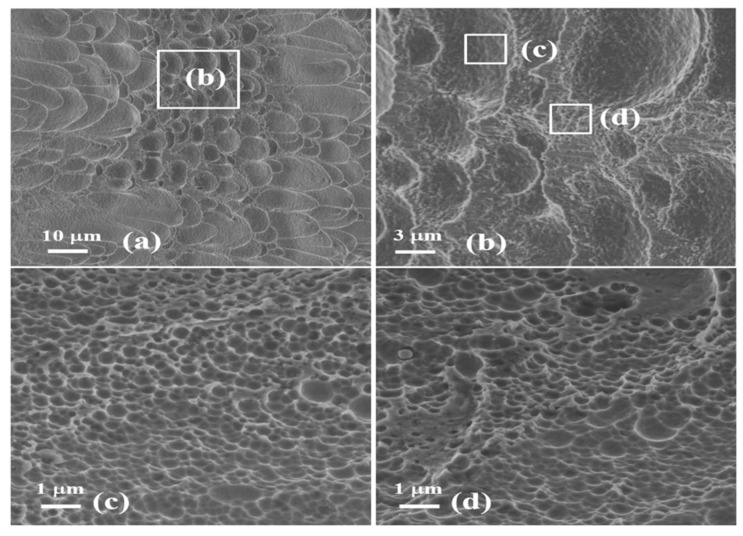
The morphology of the fracture surface in the specimen with the SCLP of 1.73. (**a**) The overall microstructural observation of the crack surface; (**b**) a high magnification of the crack surface, and the further observations with higher magnification inside (**c**) and outside (**d**) the dimple.

To study the mechanism of the shear localization fracture, the microstructure of fractured surfaces of the tested SCSs was further analyzed by SEM. Figure 7a and Figure 8a illustrate that the fracture surfaces of the SCSs with various SCLPs have same dimple structures indicating a ductile fracture mode. The dimple density increases with the SCLP increasing and the size of dimples is homogeneous in the direction vertical the shear direction but heterogeneous along the shear direction. This observation shows that different mechanisms of intergranular relative slip proceeded during the ASB formation. The highly magnified SEM fractographs show that, under the loading condition with small SCLP, the relative slip between material grains caused by shear stress results in some facets 3–5 µm in size outside the dimple (marked by white arrows in Figure 7b which represents a quasi-cleavage fracture mode. Further observations by magnified SEM demonstrate that the micro-mechanism of material fracture is quite different in different regions of fracture surface. Inside the dimples, a large temperature rise leads to a small amount of material to melt and form a very thin melting layer on the dimple surface (Figure 7c), and outside the dimple, the material is not fully melted because of the limited temperature rise and remains in the semi-molten state, i.e., the material on the fracture surface is in a micro-particle state (Figure 7d).

However, as the loadings SCLP is large, the fracture surface is characterized by the pure ductile fracture mode generated in the shear stress (Figure 8b). Both inside and outside the dimple, the larger temperature rise not only causes the material to melt, but also forms the very homogeneous fracture surfaces consisting of the submicron-scaled dimples with the average size 0.3 µm (Figure 8c,d). The implication of these observations is that, in the SDL process, the microstructural evolution of Ti6Al4V alloy is controlled by multi-scaled micro-mechanisms, that is, it is, first of all, through the nucleation, growth and cohesion of the microvoids from the submicron-scale dimples then to that of the micrometer-scale dimples, and finely from the micro-crack evolution to the fracture induced by macro-scaled cracks.

**Figure 9:**
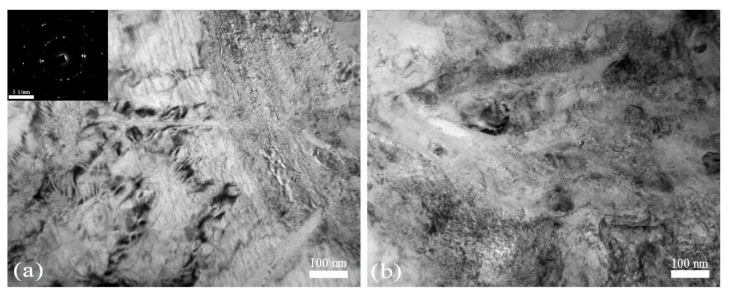
TEM images and SAED pattern of the microstructures of the SCS loading with the SCLP 0.58, (**a**) inside the ASB and (**b**) outside the ASB.

**Figure 10:**
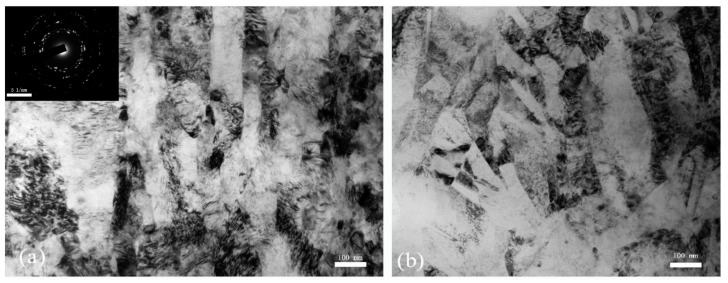
TEM images and SAED pattern of the microstructures of the SCS loading with the SCLP 1.73, (**a**) inside the ASB and (**b**) outside the ASB.

The TEM observations of the SCS microstructures are shown in Figure 9 and Figure 10. When the SCLP equals to 0.58, the TEM observations of the SCS show that the microstructure of polygon grains has developed inside the ASB (Figure 9). The size of equiaxed grains is in the region of 50–100 nm, and the average size is measured as 70 nm. The equiaxed grains in the severe SDL region have diffuse boundaries as reported in early study [26]. The SAED patterns with many rings of diffraction spots demonstrate the presence of grain boundaries with high angles of misorientations (Figure 9a). In the area of equiaxed grains, lots of the DRX grains with a range of size 20–110 nm have developed and the massive dislocation cells of high density have formed for this condition. The original coarser grains of the alloys with α + β phases gets evidently refined to form the ultra-fine grain structure. This microstructural evolution is obviously attributed to the formation of abundant dislocation slip systems and the action of the high angle misorientations stress loadings during the SDL. Away from the ASB, the structure with sufficiently refined equiaxed grains with diffused boundaries and the dislocation cells with high density are commonly observed. The plastic flow of alloy was mainly induced by the dislocation migration with the abundant slip systems in this area. A significant observation is that almost no DRX grains were developed outside the band. However, the densities of dislocation cells both inside and outside the band are approached to equal each other. This observation demonstrates that it is the nucleation and growth of the DRX grains, not the dislocation formation and movement, is the driving force for the ASB formation.

As the SCLP is 1.73, the TEM observation of the SCS microstructures and the corresponding SAED patterns are shown in Figure 10. It is apparent that, inside the ASB (Figure 10a), the structure with large coarse grains has evolved to that of the fine elongated grains without visible diffuse boundaries in this area. The SAED pattern shows that these new grain boundaries have high angles of misorientations. After severe SDL process, the microstructure is mainly composed of remnant elongated grains and local dislocation cell structure. These high density dislocations propagate and tangle inside these elongated grains. The dislocation movement is a major plastic deformation mode due to lower critical shearing stress of the alloy and the ultrafine grain structure provides more slip systems in operation than that in the coarse grain structure. This observation is in consistence with the results of Refs. [18,19]. Similarly, the DRX grains widely developed in the area of high dislocation density. The range of grains is from 20 nm to 100 nm in size. Most of the grains tangle with the dislocation and a few of grains are free of dislocations. The ring SADP indicate the characteristic of nanoscale polycrystalline materials. The formation of the DRX grains inside the ASB of the SCSs not to be broken (Figure 9a and Figure 10a) is in consistence with the results obtained by Rittel [10,11]. Far from the ASB (Figure 10b), it is clear to catch the vision of the sufficiently diffuse boundaries among the elongated grains. Similarly, a large number of dislocation cells develop outside the band. The diffuse boundaries was induced in the shear plastic deformation process.

**Figure 11:**
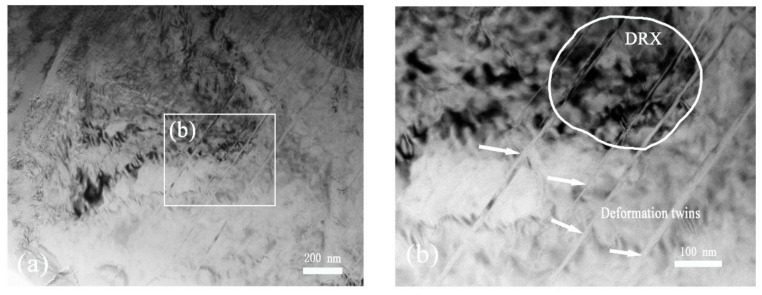
TEM images of twining microstructure of materials inside the ASB for the SCSs. (**a**) inside the ASB; (**b**) a high magnification of twining and DRX.

An unexpected result of this work is that some deformation twins presenting in the ASB are clearly observed in such specimens that were not loaded to failure (Figure 11). The observations have evident differences between the two kinds of specimens with the different SCLPs. The widths of these twin bands formed in the SDL process are changed in the range of 10–30 nm in the specimen. The DRX grains formed in the vicinity of twinning grains and thus, it can be considered that both of them control the plastic deformation localization of the alloy. It is therefore demonstrated that, similar to the formation process of the DRX grains in the SDL region, the development of twining grains is possibly to precede the ASB failure and also likely to be a dominant micromechanical factor in the process of the ASB formation [27,28].

## 4. Conclusions

The SCSs of Ti6Al4V alloy were used in the dynamic tests to investigate the micro-mechanisms for the ASB formation. The major conclusions are summarized as follows:

(1) The SDL of alloy is closely related to the SCLP of the combined shear-compressive loadings. The larger the SCLP, the more serious the SDL is.
(2) The β-phase of alloy determines the strength and hardenability of material and the bearing capacity to external loadings. It is the primary resistance to the SDL by preventing the nucleation, growth and cohesion of microvoids.
(3) In the ASB failure process, the microstructural evolution of alloy is a process characterized by multi-scale micro-mechanisms. The entire process involves the nucleation, growth and cohesion of the microvoids from the submicron-scale dimples to that of the micrometer-scale dimples, then to that of the microcracks, and finely to the ductile fracture resulting from the macroscale crack propagation.
(4) Under the combined SCL condition, there are many kinds of micro-mechanisms that affect the formation of the ASB, such as the DRX development, high density dislocation cell and dislocation migration, twins, and ultra-fined grains formation. The DRX is a main cause for the ASB formation, and however the formation of twining grains may be another major cause for the evolution of shear bands.
